# Nudge Theory: Effectiveness in Increasing Emergency Department Faculty Completion of Residency Assessments

**DOI:** 10.5811/westjem.57721

**Published:** 2023-12-06

**Authors:** Amelia Gurley, Colin Jenkins, Thien Nguyen, Allison Woodall, Jason An

**Affiliations:** Riverside Community Hospital, Department of Emergency Medicine, Riverside, California

## BACKGROUND

Assessments are a core component of residency training to assess development in the general competencies expected of all physicians.^1^ Many methods are employed to evaluate performance, from checklists to computer-based questionnaires, as no single best practice exists.^2^ Common to most, however, are barriers to the completion of assessments.^3^ For example, residents and faculty often cite a perceived lack of time to perform assessments, which may lead to suboptimal compliance in completing assessments.^3^ Some methods of assessment, such as providing narrative feedback to residents by faculty, may be seen as too burdensome.^3^ The emergency department represents an especially challenging environment to overcome these barriers given the high cognitive demand placed on faculty and residents by default.

One possible strategy to enhance faculty compliance in completing assessments is to implement behavioral nudging into social and physical environments. Borrowed from behavioral economics, nudge theory involves use of evidence-based “nudges” that incorporate positive reinforcement and indirect suggestions to influence decisions and behavior.^4^ Nudges can include use of the following: priming (environmental cues to subconsciously drive behavior); default options (desirable options are preselected as the default choice and thereby easiest for individuals to take); norm-based nudges (comparing individual behavior to peer practice); commitment (making a public promise to complete a task); and salience (drawing attention to a particular option through colors or a compelling story), among others.^4^ For instance, in the surgical intensive care unit, hand hygiene compliance was enhanced when individuals were primed with a citrus-like fragrance that was dispensed into the environment.^5^ In another example, medical student assessments were completed more often when faculty were prompted with electronic forms at the end of shifts, rather than relying on them to complete paper forms at their own discretion.^6^ In this study, we evaluated the effectiveness of two priming nudges and one norm-based nudge in increasing compliance of faculty in completing assessments of emergency medicine residents.

## OBJECTIVES

Our primary objective in the study was to assess the effectiveness of nudge interventions in increasing the number of resident performance assessments completed by attending physicians. This was assessed by comparing the number of assessments completed during the year prior to implementation of the nudge interventions with the years following their implementation. Our secondary objective was to identify which particular method was employed with the greatest frequency.

## CURRICULAR DESIGN

This project qualified as a research study conducted in established or commonly accepted educational settings. The Research Oversight Committee approved the Institutional Review Board Exempt Review Form request for exemption. The study took place at Riverside Community Hospital, a tertiary-care referral academic/community medical center in Riverside, California. The residency program at Riverside Community Hospital is a three-year emergency medicine residency accredited by the Accreditation Council for Graduate Medical Education. Each class has 13 residents per year for a total of 39 residents. We had approximately 28–30 faculty during the study, and 28 faculty received prior training on completing end-of-shift assessments.

We collected pre-intervention data from July 1, 2019–June 30, 2020 with an email link sent to faculty at the beginning of the academic year. They were sent periodic email reminders to complete the survey. The intervention started on July 1, 2020. The post-intervention data was collected from July 1, 2020–May 11, 2021.

Three primary nudges were used as the intervention to increase the number of end-of-shift assessments. We selected the nudges based on previous studies, which showed people change behavior based on social comparison.^7^ People also tend to choose the most visible option.^8^ The first nudge was to create a homepage on the faculty phone with a direct link to the end-of-shift assessment survey. The second nudge was a quick response (QR) code posted at the faculty work stations throughout the department: in the main ED; in the rapid care (lower acuity) zone; and in the faculty break room. The third nudge was based on a social proof heuristic. At the end of each block an email was sent to all faculty with the total number of assessments completed for the block, with comparisons to other faculty members’ completion rate and a link to the survey.

At the end of the study period, all faculty received a survey asking which nudge was used the most often. Faculty were asked to rank each intervention, from used most often (weighted score of 3) to least often (weighted score of 1). The survey link in the email reminder was created in Surveymonkey.com (Momentive, San Mateo, CA). We created the QR code flyer on canva.com (Surry Hills, Australia).

We believe that the interventions in this study can be replicated at many other institutions. The QR code should be posted in highly visible locations near the faculty workspace in the ED. We discovered that many faculty members required detailed instructions on how to create a homepage on their mobile devices. However, the faculty reported that once the homepage was set up, it was the easiest way to complete the assessments. The end-of-the-block summary of the total number of assessments completed by faculty may be an administrative burden to some institutions.

## IMPACT/EFFECTIVENESS

As shown in Table 1, there was a 15.8% increase in the number of assessments completed in the year after these interventions were implemented, with the number of completed assessments increasing from 3,663 (305 assessments per month) in the pre-intervention year to 4,243 (354 assessments per month) in the first post-intervention year. This increase was sustained in the following year, with 4,534 assessments (453 assessments per month) completed to date. This trend suggests that our “nudge” interventions may have been effective in producing a long-term change in faculty behavior patterns.

**Table.** Number of assessments completed over time charted against timeline of interventions.

Time frame	7/1/19–6/30/20 (pre-intervention)	7/1/20–6/20/21 (post-intervention)	7/1/21–6/1/22 (post-intervention)
Number of responses	3,663	4,243	4,534
Evaluations per month	305	354	453

When surveying the 28 faculty to determine which nudge was most effective, there was an 85.7% (24) response rate. Of the respondents, 19 (79%) indicated that their most frequently used nudge was the survey link saved onto their phone, and that they completed over 75% of their assessments this way. Thirteen respondents (54%) reported that the nudge based on social heuristics—the link at the end of the monthly emails—was the second most frequently used. Only one respondent used the QR code flyers most frequently, and 20 (83%) stated they never used the QR code at all.

From our experimental design, we learned that nudges used online could be effective in increasing completion rates of assessments. A surprising limitation was the grouping of data into certain time frames, which could be delineated in future iterations to determine the impact that time of year has on response rates. We could also compare efficacies of different interventions, such as comparing a baseline rate of using home-screen survey links only to this baseline plus an added intervention, to assess the importance of each added variable and help determine which interventions truly provide benefit.

This assessment of our interventions’ impact is limited by several factors. As the number and makeup of faculty changed during the intervention, it was not possible to determine whether a statistically significant number of faculty changed their practice as a result of this intervention. The increase in the assessment completion rate may also be due not only to our interventions but also to outside factors such as changing hospital policies, number of faculty, the impact of the COVID-19 pandemic, overall departmental shifts in attitude, or the Hawthorne effect, any of which may have played a role in influencing behavior. It is also difficult to distinguish which of the various interventions actually impacted attending behavior, as all were implemented simultaneously, and survey replies were anonymous and may be subject to recall bias. For example, it is possible that the presence of QR codes at workstations was responsible for the large increase in phone home-screen assessment completion.
